# PVT1/miR-16/CCND1 axis regulates gastric cancer progression

**DOI:** 10.1515/med-2022-0550

**Published:** 2023-01-31

**Authors:** Haidong Lv, Dixia Zhou, Guoqing Liu

**Affiliations:** Department of Tumor Surgery, Qinghai People’s Hospital, Xining 810007, Qinghai, China; Department of Tumor Surgery, Qinghai People’s Hospital, Republic Road No. 2, Xining 810007, Qinghai, China

**Keywords:** PVT1, miR-16, CCND1, gastric cancer

## Abstract

Long non-coding RNA plasmacytoma variant translocation 1 (PVT1) has been reported to be a vital modulator in tumorigenesis of gastric cancer (GC). However, the detailed regulatory mechanism of PVT1 in GC remains largely unclear. In this work, the expressions of PVT1 and microRNA-16 (miR-16) were detected by quantitative real-time PCR (qRT-PCR) in GC tissues and cell lines. GC cell lines NCI-N87 and MKN45 cell lines were chosen for the following assays. After PVT1 was overexpressed or depleted, CCK-8 and Transwell assays were performed to examine the cell viability and invasive capacity. Cell cycle was analyzed by flow cytometry. The expression of cyclin D1 (CCND1) at mRNA and protein levels was measured by qRT-PCR and western blot. The competitive endogenous RNA molecular mechanism among PVT1, miR-16 and CCND1 was verified by bioinformatics analysis, luciferase-reporter gene assay and RNA immunoprecipitation assay. In the present study, it was revealed that PVT1 expression was remarkably evaluated in GC tissues and cell lines than that in the corresponding control group. PVT1 positively regulated the proliferation, migration and cell cycle progression of GC cells. Besides, miR-16 was identified as a target of PVT1, and CCND1 was identified as a target of miR-16. The depletion of PVT1 promoted the expression of miR-16 and suppressed CCND1 expression. Moreover, either miR-16 inhibitor or CCND1 overexpression plasmid could reverse the promoting effects of PVT1 on the malignant biological behaviors of GC cells. In conclusion, PVT1 promoted CCND1 expression by negatively regulating miR-16 expression to enhance the viability, invasion and cell cycle progression of GC cells.

## 1 Introduction

Gastric cancer (GC) is a common malignancy worldwide, which is the third leading cause of cancer-related death [1]. The prognosis for patients with advanced GC stage is still poor with the median survival time of about 3 months to 1 year [2,3]. *Helicobacter pylori* (HP) and some genetic changes are involved in GC progression, and the morbidity of GC is especially high in Eastern Asia, and men are more susceptible than women [4,5]. Even though GC in the early stage can be cured by endoscopic resection or surgical operation, in some developing countries, including China, most of the patients have GC in advanced stage when they are diagnosed [6].

Long non-coding RNAs (lncRNAs) are a group of non-protein coding RNA transcripts and their length exceeds 200 nucleotides [7]. Abnormal expressions of lncRNAs contribute to tumorigenesis, cancer progression and metastasis of a lot of malignancies including GC [8–12]. For example, upregulation of lncRNA UCA1 expression level is significantly associated with lymph node metastasis and high TNM stage of GC patients, and UCA1 depletion inhibits GC cell migration and invasion abilities [10]. Also, lncRNA XIST exerts oncogenic effects by facilitating cell proliferation, migration and invasion [11]. lncRNA plasmacytoma variant translocation 1 (PVT1) has been reported as a tumor promoter in multiple malignancies such as gallbladder cancer [13] and esophageal adenocarcinoma [14]. Moreover, PVT1 has been found to be highly expressed in GC tissues and cells [15–17], while the underlying mechanism of PVT1 in GC is still poorly understood.

MicroRNAs (miRNAs) are a class of small non-coding RNA molecules (containing about 20–25 nucleotides), which are involved in RNA silencing and post-transcriptional regulation of gene expression by binding to the 3ʹ-untranslated regions (3ʹ-UTRs) of their target mRNAs [18]. Several dysregulated miRNAs have been reported to play vital roles in GC, such as miR-146a, miR-214 and so on [19–21]. microRNA-16 (miR-16) has been reported to be involved in the pathogenesis of various diseases, such as chronic lymphocytic leukemia [22] and osteosarcoma [23]. However, a more detailed mechanism of miR-16 in GC has not been clearly understood.

In this study, we mainly explored the role of PVT1 in regulating GC cell viability, migration and cell cycle progression. Herein, we report that PVT1 is highly expressed in GC, and it functions as an oncogenic lncRNA by regulating miR-16 and cyclin D1 (CCND1).

## 2 Materials and methods

### 2.1 Clinical samples collection

All GC tissues and matched adjacent normal tissues (*n* = 54) were obtained during surgery from Qinghai People’s Hospital between March 2015 and June 2019. All the patients did not receive any preoperative anti-cancer therapy including chemotherapy and radiotherapy.


**Ethics statement:** Written informed consents were obtained and this research was approved by the Ethics Committee of the Qinghai People’s Hospital (Approval No. 20190703). This study was carried out in accordance with the guidelines of the Declaration of Helsinki.

### 2.2 Cell lines

Human GC cell lines (AGS, NCI-N87, KATO III and MKN45 cells) and human gastric mucosal epithelial cell line (GES-1) were purchased from China Center for Type Culture Collection (CCTCC; Wuhan, Hubei, China). These cells were cultured in Dulbecco’s modified Eagle medium (DMEM) supplemented with 10% fetal bovine serum (FBS; Gibco, Carlsbad, CA, USA) and 1% penicillin/streptomycin (Invitrogen, Carlsbad, CA, USA) at 37°C in an incubator containing 5% CO_2_.

### 2.3 Cell transfection

PVT1 or CCND1 overexpression plasmid (PVT1 or CCND1) and the corresponding empty vector (pcDNA), small interference RNA (siRNA) targeting PVT1 (si-PVT1) or CCND1 (si-CCND1) and their negative control (si-NC), miR-16 mimic and its negative control (miR-NC), miR-16 inhibitor (in-miR-16) and its negative control (in-NC) were purchased from GenePharma Co., Ltd (Shanghai, China). Transfection of plasmids, siRNAs, miRNAs or miRNA inhibitors into NCI-N87 and MKN45 cells was performed by Lipofectamine^®^ 3000 (Invitrogen, Carlsbad, CA, USA) according to the manufacturer’s protocol.

### 2.4 Quantitative real-time PCR (qRT-PCR)

Total RNA from tissues or cells was extracted by TRIzol reagent (Invitrogen, Carlsbad, CA, USA) according to the manufacturer’s protocols. Reverse transcription was performed with a TIANScript RT Kit (Tiangen Biotech, Beijing, China). Then cDNAs were used as the template for qRT-PCR, which was completed with a SYBR^®^ Premix Dimmer Eraser kit (Takara, Dalian, China) on the CFX96 Touch™ real-time PCR detection system (Bio-Rad Laboratories, Hercules, CA, USA). The relative expression of the genes was calculated by the 2^−ΔΔCt^ method using glyceraldehyde-3-phosphate dehydrogenase (GAPDH) or U6 as internal controls.

### 2.5 Western blot

Total proteins of the cells were extracted by RIPA lysis buffer (Beyotime, Shanghai, China) and the concentration of the protein sample was detected with a BAC kit (Pierce, Rockford, IL, USA). Equal amounts of the proteins were separated by sodium dodecyl sulfate polyacrylamide gel electrophoresis and transferred onto polyvinylidene fluoride (PVDF) membranes, and then incubated for 1 h with 5% non-fat skim milk/tris buffered saline tween. Subsequently, the membranes were incubated at 4°C overnight with anti-CCND1 antibody (ab134175; Abcam, Cambridge, UK, 1:500) and anti-GAPDH antibody (ab181602; Abcam, Cambridge, UK, 1:2,000), respectively, followed by incubation with the secondary antibody (1:2,000; Boaosen Biotechnology, Beijing, China) for 2 h at room temperature. Finally, the protein bands on the PVDF membranes were developed by using an enhanced chemiluminescence kit (Pierce, Rockford, IL, USA) according to the manufacturer’s protocol.

### 2.6 Cell Counting Kit-8 (CCK-8) assay

The cell viability was detected by CCK-8 (Dojindo Laboratories, Tokyo, Japan) according to the manufacturer’s instructions. Briefly, the GC cells were inoculated to 96-well plates (about 1,000 cells per well) and cultured, and 10 μL of CCK-8 solution was added to each well at 0, 24, 48 and 72 h, and then the cells were incubated with CCK-8 solution for 1 h. Next, the absorbance of the cells was detected at 450 nm with a microplate reader (BioTek, Winooski, VT, USA).

### 2.7 Cell invasion assay

Transfected NCI-N87 and MKN45 cells, suspended in serum-free DMEM, were transferred into the upper chamber of Transwell insert with 8 μm pore size polycarbonate membrane (Corning, NY, USA), which was covered with a layer of Matrigel. The bottom chamber of the insert was filled with complete medium containing 10% FBS. Then the cells were cultured. After 24 h, the cells on the surface of the upper membrane were carefully removed. Invasive cells attached to the lower surface were fixed by 95% ethanol and stained with 0.2% crystal violet solution (Sigma Aldrich, Milwaukee, WI, USA). Ultimately, the cells were counted by an inverted microscope. The cells in five randomly selected visual fields of the membrane were counted.

### 2.8 Flow cytometry

In each sample, a total of 10^6^ cells were fixed by 75% ethanol overnight in an ice bath. Subsequently, the cells were washed with phosphate buffer saline and centrifuged, followed by resuspension with 100 μL of RNase A solution and incubation at 37°C for 30 min. Next, propidium iodide staining solution was added to the cell suspension, followed by incubation for 30 min at 4°C. Next, cell cycle analysis was performed with a flow cytometer (Becton Dickinson, Heidelberg, Germany).

### 2.9 Luciferase-reporter gene assays

The mutant and wild-type fragments of PVT1 or CCND1 3ʹ-UTR containing miR-16 binding sites was constructed and cloned into pmirGLO reporter vectors (Promega, Madison, WI, USA). These recombinant reporter vectors were then transfected into GC cells together with miR-16 mimics, miR-NC, in-NC or in-miR-16. The relative luciferase activity was measured with a dual luciferase assay kit (Promega, Madison, WI, USA) at 48 h after transfection.

### 2.10 RNA immunoprecipitation (RIP) assay

To verify the direct interaction between PVT1 and miR-16, RIP assay was performed using an EZ-Magna RIP Kit (Millipore, Billerica, MA, USA) according to the manufacturer’s instruction. NCI-N87 and MKN45 cells were lysed using RIP lysis buffer containing protease inhibitor and RNase inhibitor, followed by the incubation with RIP buffer containing magnetic beads conjugated with human anti-argonaute 2 (anti-Ago2) antibody (Millipore, Billerica, MA, USA) or negative control normal mouse IgG (Millipore, Billerica, MA, USA) for 1 h. Subsequently, after protein removal with proteinase K, the immunoprecipitated RNA was extracted for qRT-PCR analysis.

### 2.11 Statistical analysis

All of the experiments were performed in triplicate, and repeated for at least three times. SPSS 20.0 (IBM, Chicago, IL, USA) was used to perform statistical analysis. D’Agostino & Pearson test was conducted to analyze the normality of the data. The data in the present work were shown as “mean ± standard deviations (SD).” Student’s *t*-test and one-way analysis of variance (ANOVA) were applied to determine the significance of differences between/among groups. The association between the pathological characteristics and the patients was analyzed by Chi-square test. *P* < 0.05 was defined to be statistically significant.

## 3 Results

### 3.1 PVT1 was highly expressed in GC tissues and cell lines

**Figure 1:**
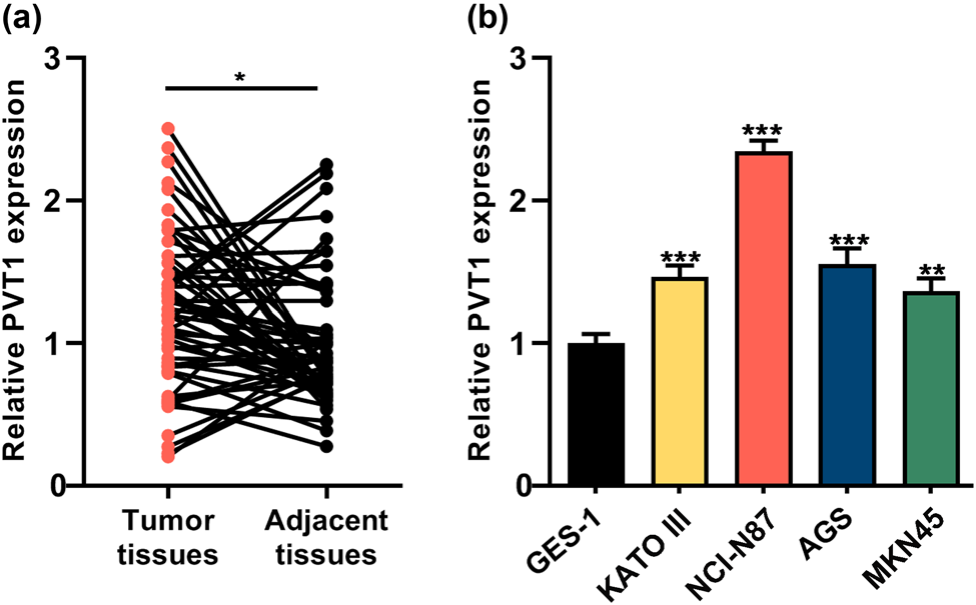
PVT1 was highly expressed in GC tissues and cell lines. (a) The expression of PVT1 in GC tissues (*n* = 54) and adjacent normal tissues (*n* = 54) was assessed by qRT-PCR. (b) The expression of PVT1 in human normal gastric mucosal cell line (GES-1) and four GC cell lines (AGS, NCI-N87, KATO III and MKN45 cells) was analyzed by qRT-PCR. All of the experiments were performed at least in triplicate. *P < 0.05, **P < 0.01 and ***P < 0.001.

First, to explore the expression characteristics of PVT1 in GC, 54 pairs of GC tissues and adjacent normal tissues were collected, and the expression of PVT1 was examined by qRT-PCR. It was revealed that, PVT1 expression was significantly increased in GC tissues than that in adjacent normal tissues (*P* < 0.05, Figure 1a). Notably, high PVT1 expression was associated with larger tumor size and lymph node metastasis of the patients (*P* < 0.05), but not associated with age, gender, HP infection and T stage of the patients (*P >* 0.05, Table 1). Additionally, the expression level of PVT1 was markedly elevated in GC cell lines (AGS, NCI-N87, KATO III and MKN45) compared with that in GES-1 cell line (*P* < 0.01, Figure 1b).

**Table 1:** Relationship between PVT1 expression and clinicopathological characteristics of GC patients

Characteristics	Number of patients	PVT1 expression		*P-*value
		Low (27)	High (27)	
Age (years)				
<50	19	11	8	0.731
≥50	35	16	19	
Gender				
Male	33	16	17	0.780
Female	21	11	10	
HP infection				
Yes	32	18	14	0.268
No	22	9	13	
Tumor size (cm)				
≥5	24	8	16	0.028*
<5	30	19	11	
T stage				
T1–T2	25	15	10	0.172
T3–T4	29	12	17	
Lymph node invasion				
Negative	16	12	4	0.017*
Positive	38	15	23	

Note: *P < 0.05.

### 3.2 Silencing PVT1 inhibited the proliferation and invasion of GC cells

**Figure 2:**
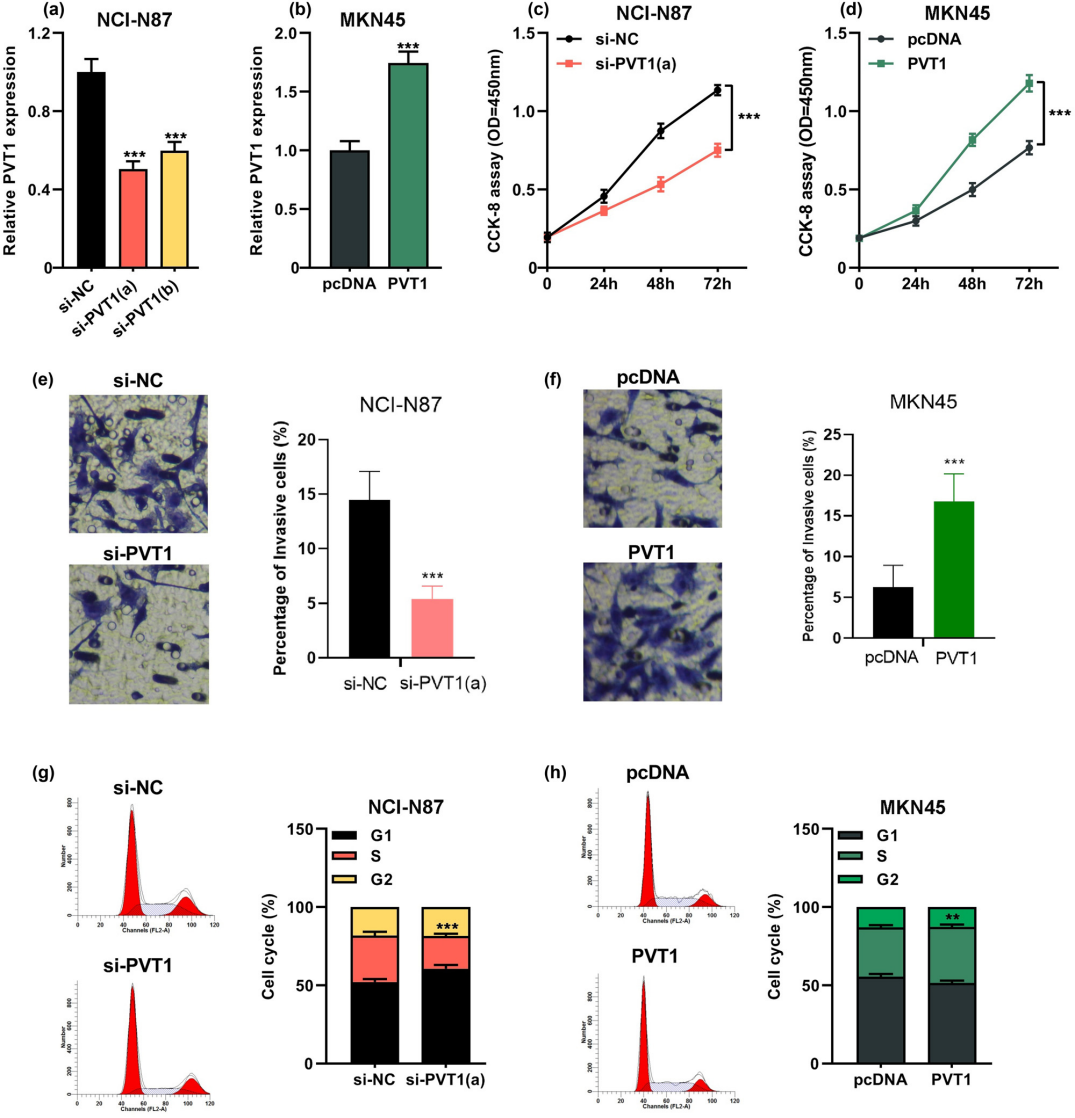
PVT1 knockdown inhibited the proliferation and invasion of GC cells, NCI-N87 cells were transfected with small interference RNA (siRNA) targeting PVT1 (si-PVT1) or negative control (si-NC). MKN45 cells were transfected with pcDNA3.1 empty vector (pcDNA) or pcDNA-PVT1 (PVT1). (a and b) qRT-PCR was used to detect the transfection efficiency at 48 h after transfection. (c and d) CCK-8 assay was performed to evaluate the viability at 0, 24, 48 and 72 h. (e and f) Transwell invasion assay was performed to assess the invasion ability of the cells. (g and h) Flow cytometry was applied to analyze the cell cycle distribution of the transfected GC cells at 48 h after transfection. All of the experiments were performed at least in triplicate. **P < 0.01 and ***P < 0.001.

Next, we further investigated the biological function of PVT1 in GC development. As shown in Figure 2a, PVT1 expression was significantly knocked down by two siRNAs in NCI-N87 cells (*P* < 0.001). Besides, the transfection of PVT1 overexpression plasmid remarkably upregulated the expression of PVT1 in MKN45 cells compared with the control group (*P* < 0.001, Figure 2b). The results of CCK-8 assay showed that PVT1 silence significantly reduced proliferation capacity of NCI-N87 cells, while PVT1 overexpression notably facilitated the proliferation of MKN45 cells (*P* < 0.001, Figure 2c and d). Moreover, a notable reduction of invasion ability was found in NCI-N87 cells with PVT1 depletion (*P* < 0.001, Figure 2e). However, PVT1 overexpression led to obvious increase in the invasion capability of MKN45 cells (*P* < 0.001, Figure 2f). Furthermore, NCI-N87 cells with PVT1 depletion showed higher percentages in the G0/G1 phase and lower percentages in S and G2 phases compared to the si-NC group (*P* < 0.001, Figure 2g). In contrast, PVT1 overexpression resulted in opposite effect on MKN45 cells (*P* < 0.01, Figure 2h). Collectively, these data suggested that PVT1 was involved in the development of GC.

### 3.3 MiR-16 was directly regulated by PVT1 in GC cells

**Figure 3:**
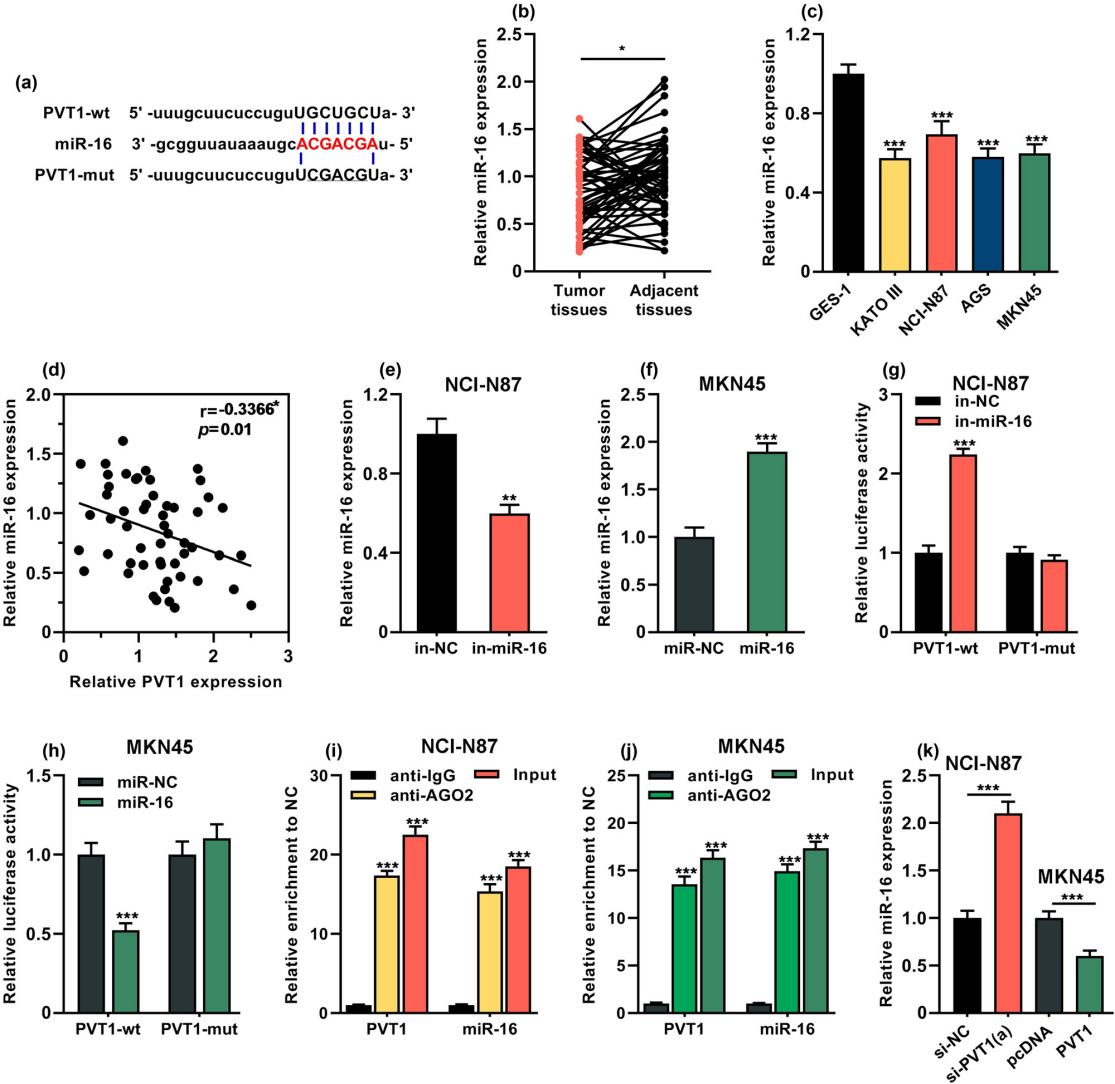
MiR-16 was directly regulated by PVT1 in GC cells. (a) The binding sites of miR-16 on PVT1. (b) qRT-PCR was performed to detect miR-16 expression in GC tissues (*n* = 54) and adjacent normal tissues (*n* = 54). (c) qRT-PCR was performed to detect miR-16 expression in GES-1 cells and four GC cell lines (AGS, NCI-N87, KATO III and MKN45). (d) Pearson’s correlation analysis was applied to assess the correlation between PVT1 expression and miR-16 expression in GC tissues. (e–h) NCI-N87 cells were transfected with miR-16 inhibitor (in-miR-16) and control (in-NC). MKN45 cells were transfected with miR-16 mimic (miR-16) or miRNA control (miR-NC). (e and f) qRT-PCR was performed to detect miR-16 expression at 48 h after transfection. (g and h) Luciferase-reporter gene assay was performed to detect the luciferase activities of reporter vectors at 48 h post-transfection. (i and j) RIP assay was performed to detect the interaction between PVT1 and miR-16. (k) qRT-PCR assay was performed to detect miR-16 expression in NCI-N87 cells with PVT1 knockdown, and in MKN45 cells with PVT1 overexpression. All of the experiments were performed at least in triplicate. *P < 0.05, **P < 0.01 and ***P < 0.001.

Next, the potential miRNAs targeted by PVT1 were predicted by StarBase database (http://starbase.sysu.edu.cn/index.php). Interestingly, it was revealed that miR-16 had a complementary binding site with PVT1 (Figure 3a). It is reported that the miR-16 family functions as a modulator of the cell cycle by targeting the genes important for the G1–S transition [24]. So miR-16 was selected as the candidate miRNA target for PVT1. qRT-PCR assay showed that miR-16 expression was remarkably decreased in both GC tissues (*P* < 0.05, Figure 3b) and cell lines (*P* < 0.001, Figure 3c). Moreover, a negative relationship between PVT1 expression and miR-16 expression was observed in 54 cases of GC tissue samples (*R* = −0.3366, *P =* 0.01, Figure 3d). Next, we transfected miR-16 inhibitor (in-miR-16) and control (in-NC) into NCI-N87 cells while miR-16 mimic (miR-16) and corresponding control (miR-NC) into MKN45 cells. qRT-PCR was used to assess the transfection efficiency and the results showed that miR-16 inhibitor led to marked reduction of miR-16 expression in NCI-N87 cells (*P* < 0.01, Figure 3e). Conversely, miR-16 expression was notably increased in MKN45 cells with miR-16 mimic transfection (*P* < 0.001, Figure 3f). Subsequently, the luciferase reporter gene assay was carried out to verify the targeting relationship between PVT1 and miR-16. As shown in Figure 3g, the relative luciferase activity of vector containing wild-type PVT1 (PVT1-wt) was markedly enhanced in NCI-N87 cells after the transfection of miR-16 inhibitor (*P* < 0.001). In contrast, the transfection of miR-16 mimic markedly reduced the luciferase activity of vector containing PVT1-wt in MKN45 cells (*P* < 0.001, Figure 3h). Importantly, no significant effect was found in that of vector-carrying mutant PVT1 (PVT1-mut) in both NCI-N87 and MKN45 cells (*P* > 0.05, Figure 3g, h). Consistently, the results of RIP assay showed that PVT1 and miR-16 were both significantly enriched in immunoprecipitates in anti-Ago2 group compared to anti-IgG group of both NCI-N87 and MKN45 cells (*P* < 0.001, Figure 3i, j), indicating that PVT1 and miR-16 interacted with each other directly. In addition, the depletion of PVT1 remarkably elevated the expression level of miR-16 in NCI-N87 cells but overexpression of PVT1 markedly decreased that in MKN45 cells (*P* < 0.001, Figure 3k). These data above suggested that miR-16 was directly and negatively regulated by PVT1 in GC.

### 3.4 CCND1 was a target of miR-16

**Figure 4:**
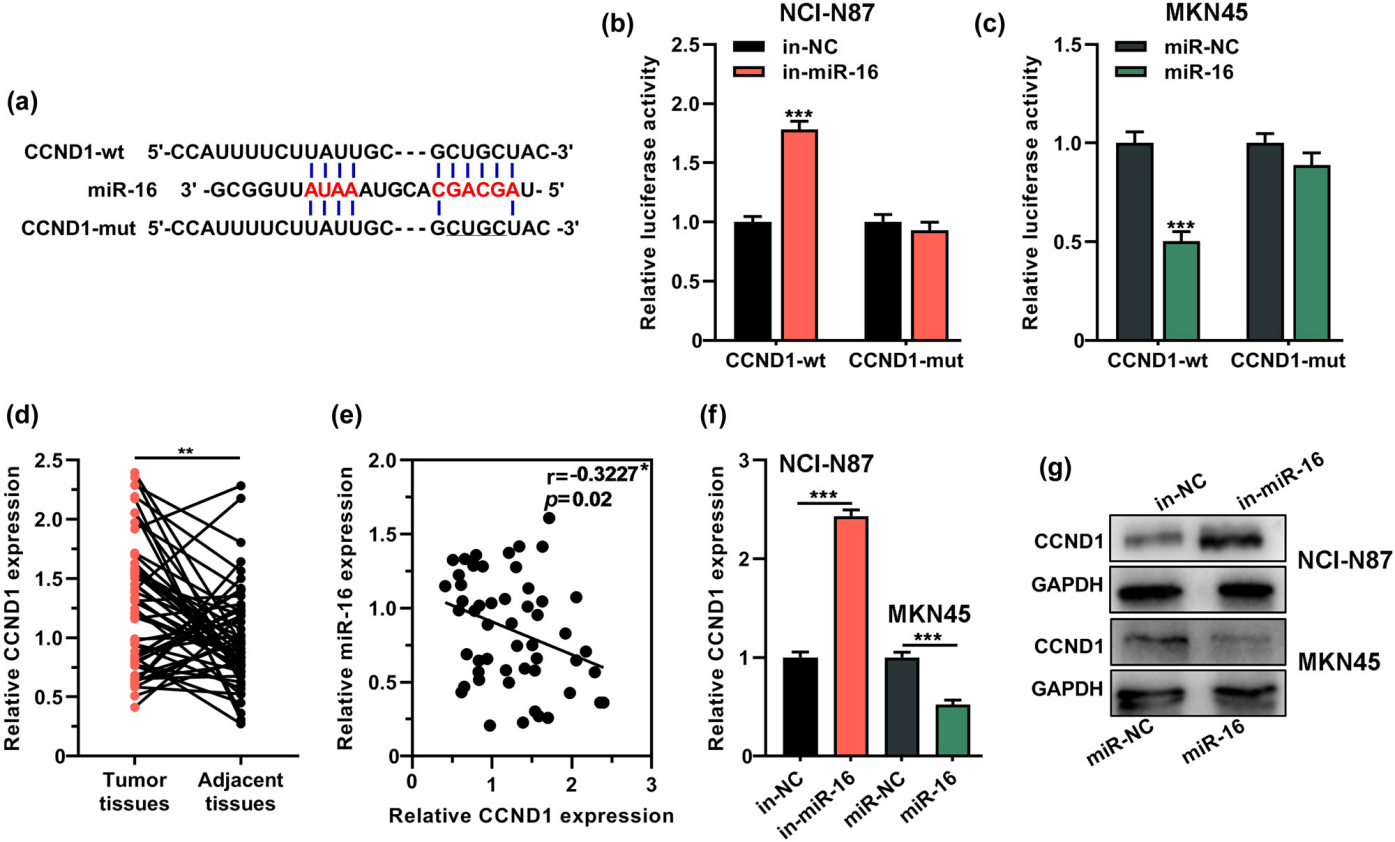
CCND1 was a target of miR-16. (a) Online database was used to predict the binding site between miR-16 and CCND1 3′-UTR. (b and c) Luciferase-reporter gene assay was performed to detect the luciferase activities of reporter vectors containing CCND1-wt or CCND1-mut at 48 h post-transfection. (d) qRT-PCR assay was performed to detect CCND1 expression in GC tissues (*n* = 54) and adjacent normal tissues (*n* = 54). (e) Pearson’s correlation analysis was applied to assess the correlation between miR-16 expression and CCND1 expression in GC tissues. (f and g) qRT-PCR and western blot assay were used to measure CCND1 expression in NCI-N87 and MKN45 cells transfected with in-miR-16/in-NC and miR-16 mimic/miR-NC, respectively. All of the experiments were performed at least in triplicate. **P < 0.01 and ***P < 0.001.

**Figure 5:**
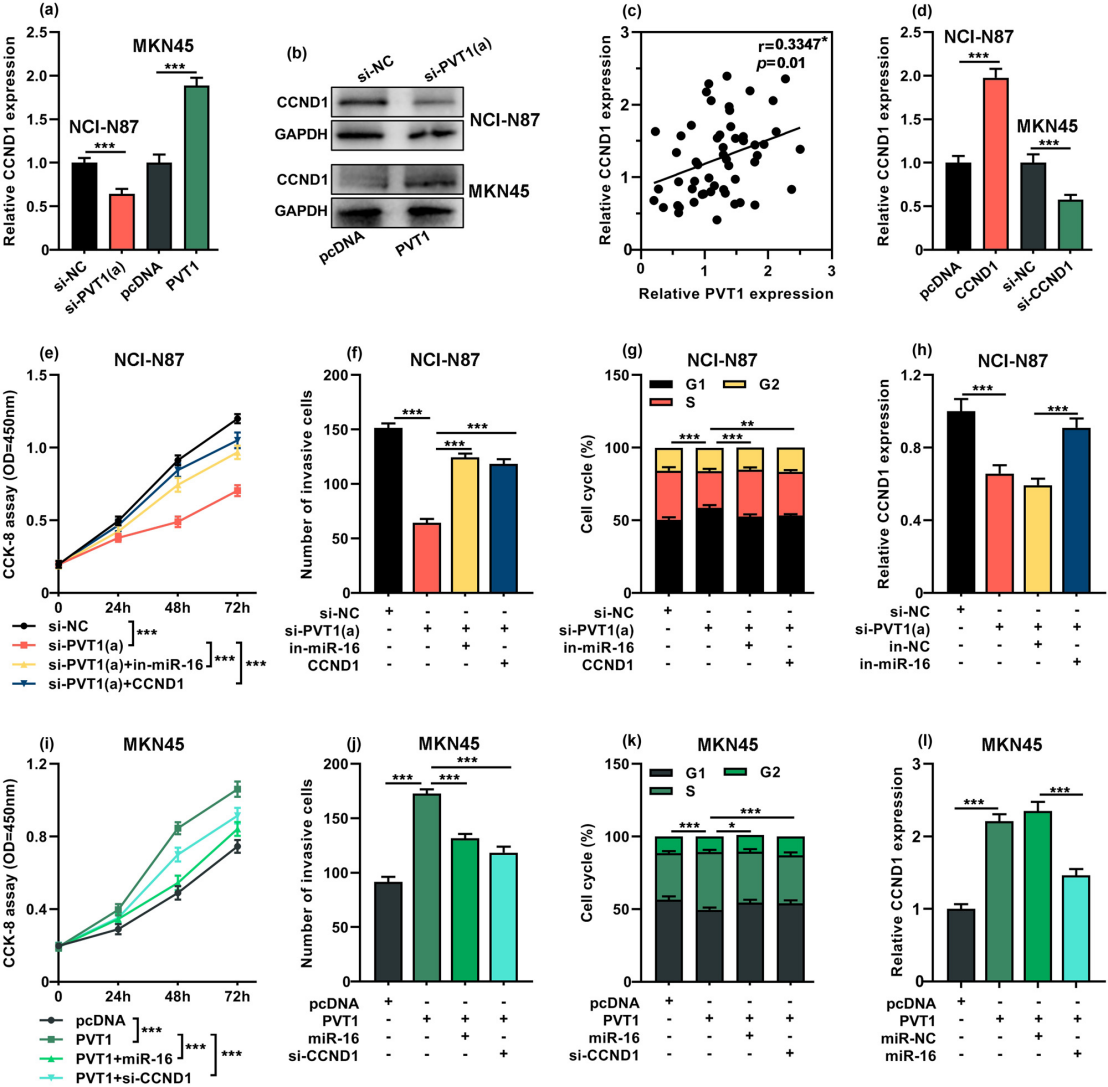
PVT1 promoted GC progression by regulating miR-16/CCND1. (a and b) qRT-PCR and western blot were used to measure the expression of CCND1 in NCI-N87 cells with transfection of si-NC/si-PVT1, and in MKN45 cells with transfection of pcDNA/PVT1. (c) Pearson’s correlation analysis was applied to assess the correlation between PVT1 expression and CCND1 expression in GC tissues (*n* = 54). (d) qRT-PCR was applied to detect the transfection efficiency of CCND1 overexpression plasmid into NCI-N87 cells or si-CCND1 into MKN45 cells. (e–g) NCI-N87 cells were transfected with si-NC, si-PVT1, si-PVT1 + miR-16 inhibitor (in-miR-16) or si-PVT1 + pcDNA-CCND1 (CCND1). (e) CCK-8 assay was performed to evaluate the cell viability. (f) Transwell invasion assay was applied to detect invasion ability. (g) Flow cytometry was carried out to assess the cell cycle distribution at 48 h after transfection. (h) qRT-PCR was performed to measure the CCND1 expression in NCI-N87 cells transfected with si-NC, si-PVT1, si-PVT1 + in-NC or si-PVT1 + in-miR-16. (i–k) MKN45 cells were transfected with pcDNA, pcDNA-PVT1 (PVT1), pcDNA-PVT1 + miR-16 mimic (miR-16) or pcDNA-PVT1 + si-CCND1. (i) CCK-8 assay was performed to evaluate the cell viability. (j) Transwell invasion assay was applied to detect invasion ability. (k) Flow cytometry was carried out to assess the cell cycle distribution at 48 h after transfection. (l) qRT-PCR was performed to measure the CCND1 expression in MKN45 cells after transfected with pcDNA, pcDNA-PVT1, pcDNA-PVT1 + miR-NC or pcDNA-PVT1 + miR-16 mimic. All of the experiments were performed at least in triplicate. *P < 0.05, **P < 0.01 and ***P < 0.001.

Subsequently, online bioinformatics databases including miRDB, miRanda and TargetScan 7.2 were searched to predict the potential downstream target mRNAs of miR-16. It was revealed that, CCND1, which was reported to be a modulator in tumor progression via promoting cell cycle progression [25], was among the candidates. Therefore, CCND1 was chosen as the target gene and the binding site between miR-16 and CCND1 3ʹ-UTR is shown in Figure 4a. Besides, the luciferase activity in NCI-N87 cells was increased after cells were co-transfected with miR-16 inhibitor and wild-type CCND1 reporter (CCND1-wt) (*P* < 0.001, Figure 4b). Meanwhile, the luciferase activity of vector containing CCND1-wt was reduced in MKN45 cells transfected with miR-16 mimics (*P* < 0.001, Figure 4c). Additionally, no significant changes were observed in that of the reporter vector containing mutant CCND1 (CCND1-mut) (*P >* 0.05, Figure 4b and c). These data suggested that miR-16 could interact with CCND1 3ʹ-UTR. Additionally, a notable increase of CCND1 expression was found in GC tissues compared with adjacent normal tissues (*P* < 0.01, Figure 4d). Besides, it was observed that CCND1 expression was negatively correlated with miR-16 expression in GC tissue samples (*R* = −0.3227, *P* = 0.02, Figure 4e). Moreover, the transfection of miR-16 inhibitor markedly facilitate mRNA and protein expression levels of CCND1 in NCI-N87 cells, while the transfection of miR-16 mimic resulted in a remarkable reduction of those in MKN45 cells (*P* < 0.001, Figure 5f and g). Collectively, the results above implied that CCND1 was a target of miR-16 in GC.

### 3.5 PVT1 promoted GC progression by regulating miR-16/CCND1 axis

Considering that PVT1 could regulate the expression of miR-16, and CCND1 was a target of miR-16, it was supposed that PVT1 promoted GC progression by targeting miR-16/CCND1 axis. First, we found that the depletion of PVT1 resulted in a significant reduction in CCND1 expression while overexpression of PVT1 markedly promoted CCND1 expression (*P* < 0.001, Figure 5a and b). Furthermore, it was revealed that the expression level of CCND1 in CG tissues was positively correlated with that of PVT1 (*R* = 0.3347, *P =* 0.01, Figure 5c). Subsequently, qRT-PCR was applied to detect the transfection efficiency of CCND1 overexpression plasmid or si-CCND1. The results showed that CCND1 expression was notably evaluated after CCND1 overexpression plasmid transfection in NCI-N87 cells, while si-CCND1 resulted in a remarkable decrease of CCND1 expression in MKN45 cells (*P* < 0.001, Figure 5d). Next, “rescue” experiments were performed. It was revealed that PVT1 depletion led to a decrease in cell proliferation (*P* < 0.001, Figure 5e) and invasive ability (*P* < 0.001, Figure 5f). Moreover, the cell cycle analysis revealed that silencing PVT1 resulted in cell cycle arrest at the G0/G1 phase (*P* < 0.001, Figure 5g). However, miR-16 inhibitor or CCND1 overexpression notably reversed these effects in NCI-N87 cells (*P* < 0.001, Figure 5e–g). Besides, miR-16 inhibition could abolish the decreased CCND1 expression triggered by PVT1 depletion (*P* < 0.001, Figure 5h). Additionally, we found that overexpression of PVT1 promoted the proliferation, invasion capability and cell cycle progression of MKN45 cells (*P* < 0.001, Figure 5i–k). However, miR-16 overexpression and CCND1 depletion could counteract these effects in MKN45 cells (*P* < 0.001, Figure 5i–k). PVT1 overexpression markedly increased the CCND1 expression, which was then reduced by the co-transfection of miR-16 mimics (*P* < 0.001, Figure 5l). These results indicated that PVT1 could modulate CCND1 expression by targeting miR-16, to regulate the progression of GC.

## 4 Discussion

GC is one of the most common malignancies. Increasing evidence indicate that lncRNAs are the promising markers/targets for GC diagnosis and treatment [26]. Increasing evidence supports that PVT1 acts as an oncogene and regulates the phenotypes of cancer cells in multiple cancers [27,28]. Additionally, PVT1 expression is correlated with shorter overall survival of the patients with head and neck squamous cell carcinoma [29] and prostate cancer [30]. In our research, the data showed that PVT1 expression was significantly evaluated in GC tissues and cell lines. Functional experiments showed that knockdown of PVT1 markedly suppressed the proliferation and invasion of GC cells and induced cell cycle arrest. These findings are in line with the previous studies [15–17,31].

Emerging studies have shown that many miRNAs are abnormally expressed in multiple malignancies including in GC [32]. miR-16-5p expression was reduced in chordoma and it suppresses cancer cell proliferation and invasion via targeting Smad3 [33]. It is also reported that miR-16 dysregulation in hepatic stellate cells (HSCs) leads to guanine nucleotide-binding α-subunit 12 (Gα12) overexpression, which activates HSCs by promoting autophagy to promote the pathogenesis of liver fibrosis [34]. In our study, miR-16 expression was observed to be downregulated in GC tissues. Increasing evidence reports that lncRNA functions as the miRNA sponge competitive endogenous RNA (ceRNA), which decreases the availability of miRNA and modulates post-transcriptional regulation of gene expression [35]. In this study, we further found that PVT1 could act as a molecular sponge of miR-16, and PVT1 could negatively regulate the expression of miR-16 in GC cells. These findings partly explain the mechanism of miR-16 dysregulation in GC.

CCND1 functions as a vital regulator of cell cycle progression by activating the G1/S transition, which results in the increase of cell proliferation and growth [36]. Mutations, amplification and overexpression of CCND1 are frequently detected in tumors, and the dysfunction of CCND1 contributes to tumorigenesis [37]. For example, high CCND1 expression is correlated with poor prognosis of the patients with GC [38]. Additionally, CCND1 is also highly expressed in hepatocellular carcinoma tissues, and it is reported that LINC00152 regulates cancer cell proliferation by miR-193a/b-3p/CCND1 axis [39]. In our study, CCND1 was also found to be significantly overexpressed in GC tissues. In addition, CCND1 was identified as a target of miR-16, and it was positively regulated by PVT1. Moreover, rescue assays showed that PVT1 could positively regulate CCND1 expression in GC cells, which was abolished by the restoration of miR-16. Furthermore, CCND1 knockdown or miR-16 overexpression could reverse the effects of PVT1 on cell proliferation, invasion and cell cycle progression. These results indicated that PVT1 was involved in GC development by regulating the miR-16/CCND1 axis.

To conclude, this study reports that PVT1 is highly expressed in GC. Besides, PVT1 may promote GC cell proliferation, invasion and cell cycle progression via regulating miR-16/CCND1 axis, indicating that PVT1 might be a potential therapy target for GC. Notably, an lncRNA may modulate multiple downstream target miRNAs, and a miRNA may regulate multiple downstream target genes. This suggests that PVT1 may be involved in a ceRNA network consisting of multiple molecules. In the following studies, the other downstream miRNAs/target genes remain to be screened out and validated.
